# Structural Racism in Healthcare: Nurses' Ethical and Professional Responsibility to Lead Structural Reform

**DOI:** 10.1002/nop2.70754

**Published:** 2026-08-26

**Authors:** Chanchal Kurup, Deepa Rijal

**Affiliations:** ^1^ School of Nursing, Midwifery and Social Sciences Norman Gardens Queensland Australia

**Keywords:** culturally safe care, governance accountability, healthcare inequity, nursing leadership, organisational reform, patient safety, structural advocacy, structural racism, workforce equity

## Abstract

**Background:**

Structural racism remains a pervasive and deeply embedded determinant of healthcare inequity globally. Despite strengthened professional standards and policy commitments to equity, racialised populations continue to experience substantial disparities in healthcare access, quality, safety and outcomes. These inequities are sustained not only through interpersonal encounters but also through structural conditions embedded within healthcare systems, workforce arrangements, governance processes and organisational cultures. Structural racism additionally affects nurses' wellbeing, workforce sustainability, psychological safety and professional progression, creating implications for both patient outcomes and healthcare system functioning.

**Aim:**

To articulate an evidence‐informed position that addressing structural racism in healthcare is a moral, ethical and professional responsibility of nurses, and to propose a conceptual framework positioning nurses as leaders of structural reform, governance accountability and equity‐driven healthcare transformation.

**Design:**

Position paper drawing on interdisciplinary empirical evidence, ethical frameworks, workforce literature and contemporary nursing scholarship.

**Discussion:**

This paper introduces the NSERF, which conceptualises structural racism as a multidimensional patient safety, workforce sustainability and governance accountability issue requiring coordinated nurse‐led structural advocacy and institutional reform. Structural racism is shown to operate through interconnected systems including healthcare policy, workforce structures, organisational governance, clinical practice and professional hierarchies, contributing to inequitable access to care, workforce exclusion, reduced psychological safety, burnout and poorer health outcomes. While nursing advocacy has historically focused on individual patient protection, this paper argues that structural advocacy is necessary to address systemic inequities embedded within institutional systems. Strategic reform directions include governance accountability, equity‐integrated clinical governance, workforce equity, structural competency education and policy engagement.

**Conclusion:**

Structural racism compromises healthcare equity, patient safety, workforce sustainability and organisational trust. Nurses possess an ethical mandate, professional authority and practice‐informed insight to lead structural reform. Moving beyond interpersonal advocacy towards sustained structural advocacy is essential for developing equitable, culturally safe, accountable and sustainable healthcare systems.

## 1 Introduction

Healthcare systems are frequently positioned as institutions grounded in equity, justice and beneficence. Yet a growing body of evidence demonstrates that structural racism continues to shape healthcare delivery, influencing access, quality, clinical decision‐making, workforce experiences and health outcomes across populations (Braveman et al. 2022; Kisa and Kisa 2025). Structural racism functions through interconnected institutional systems that differentially distribute resources, opportunities, authority and risks along racial lines, reinforcing inequities across healthcare, employment, education and broader social structures (Williams et al. 2019).

This position paper is written primarily for nurse leaders, nurse educators, policymakers and healthcare organisations seeking practical guidance on translating evidence about structural racism into nurse‐led structural reform. While an extensive literature has documented the existence and health consequences of structural racism, comparatively few papers articulate an integrated, actionable framework through which nurses can lead structural change. This paper addresses that gap by advancing a novel, nurse‐led framework that translates evidence into concrete governance, workforce, education and policy strategies, distinguishing this contribution from existing descriptive and conceptual accounts of structural racism in healthcare.

The relationship between structural racism and adverse health outcomes is extensive and increasingly well established. Kisa and Kisa (2025) scoping review identified consistent associations between structural racism and poorer outcomes across maternal and infant mortality, cardiovascular disease, cancer, HIV care, mental health and infectious disease outcomes. Similarly, Hamed et al. (2022) demonstrated that racialised minority populations frequently experience barriers to healthcare access, dismissal of symptoms, inequitable treatment and reduced quality of care. During the COVID‐19 pandemic, racialised populations experienced disproportionately poorer outcomes globally, further exposing how structural inequities shape vulnerability, healthcare access and system responsiveness.

Importantly, structural racism also carries substantial consequences for healthcare professionals and workforce sustainability. Racialised nurses frequently report exclusionary workplace cultures, inequitable progression opportunities, emotional exhaustion, professional isolation and reduced psychological safety (Mostafa et al. 2025). These conditions directly undermine nurses' psychological wellbeing and resilience and are strongly associated with reduced retention and workforce sustainability, underscoring that structural racism is a workforce imperative and not solely a patient‐safety concern.

These experiences contribute to burnout, workforce attrition, underutilisation of expertise and moral distress, affecting both individual wellbeing and organisational capability. Structural racism must therefore be understood not solely as a patient inequity issue, but also as a workforce sustainability and governance accountability issue.

Although healthcare systems increasingly acknowledge inequity, responses often remain fragmented, localised and insufficiently embedded within organisational governance and policy reform (Kisa and Kisa 2025). Current approaches frequently emphasise interpersonal cultural competence or descriptive inequity reporting while neglecting the institutional mechanisms through which inequities are reproduced and sustained. This gap elevates the need for credible professional leadership capable of translating evidence into measurable structural reform.

Nurses occupy a unique position within healthcare systems due to their sustained proximity to patients, communities, workforce systems and organisational processes. As the largest healthcare profession globally, nurses maintain direct visibility of how inequities shape both patient care and workforce experiences across the continuum of care (International Council of Nurses 2023). However, nursing advocacy has historically been conceptualised primarily as individual patient advocacy rather than structural reform (Pitcher et al. 2025).

While interpersonal advocacy remains essential, it is insufficient to dismantle inequities embedded within institutional systems and policy environments.

This paper argues that structural racism should no longer be conceptualised solely as a diversity or social justice issue within healthcare, but rather as a multidimensional patient safety, workforce sustainability and governance accountability issue requiring coordinated institutional action. To address this gap, the paper introduces the Nurse‐Led Structural Equity Reform Framework (NSERF), which positions nurses not only as patient advocates but also as leaders of governance accountability, workforce reform and equity‐driven healthcare transformation.

## 2 Conceptual Framework: NSERF

**FIGURE 1:**
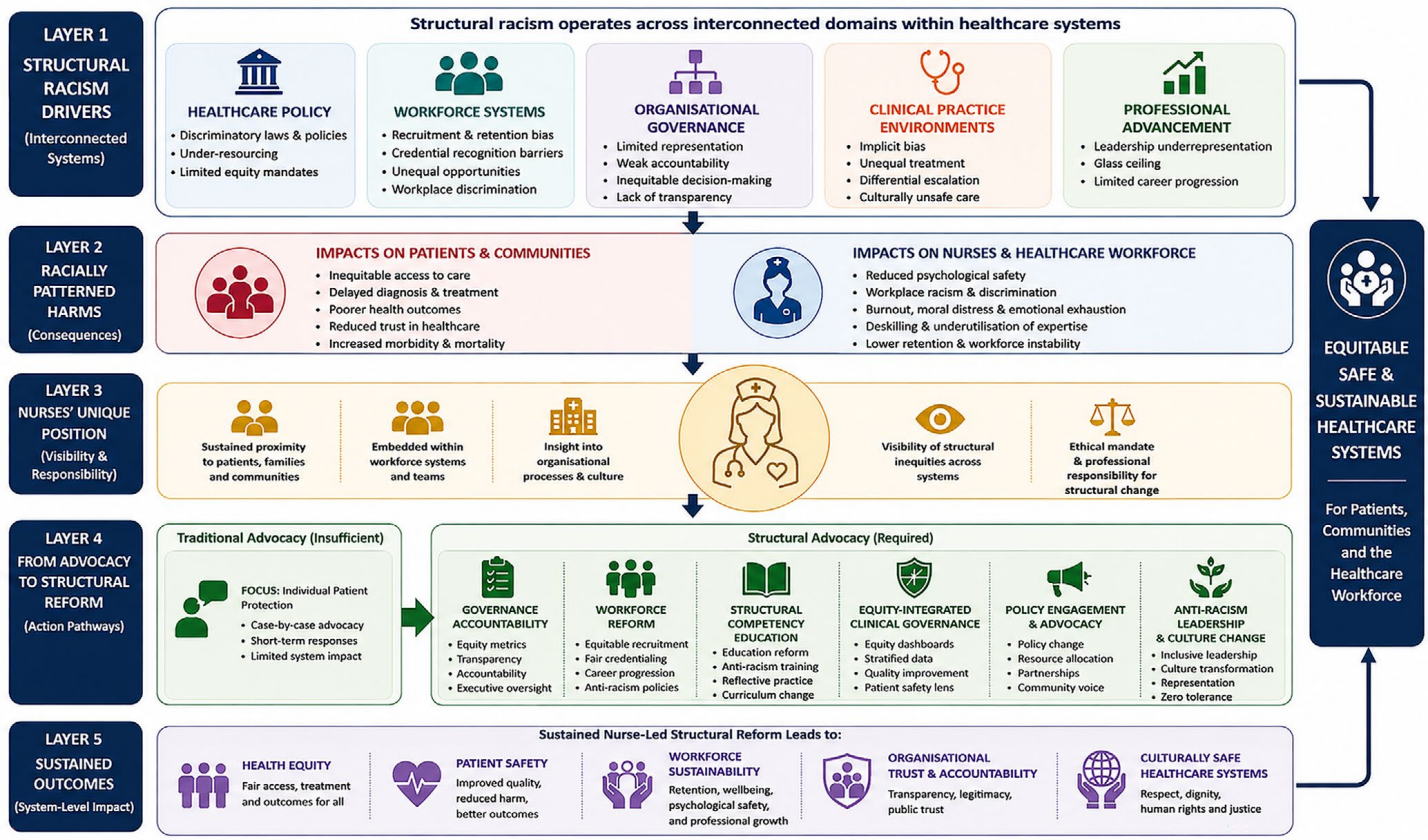
Nurse‐Led Structural Equity Reform Framework (NSERF).

This paper introduces the NSERF as a conceptual model synthesising the relationships between structural racism, healthcare inequities, workforce consequences and nurse‐led structural reform within healthcare systems. Figure 1 presents the NSERF and illustrates how interconnected structural inequities contribute to racially patterned harms affecting both patients and healthcare professionals, while also demonstrating how nurses can act as catalysts for governance accountability, workforce reform and equity‐driven system transformation.

The framework positions structural racism as an interconnected systems‐level phenomenon operating across healthcare policy, organisational governance, workforce structures, clinical practice and professional advancement pathways. These interconnected inequities contribute to racially patterned harms affecting both patients and healthcare professionals, including inequitable access to care, reduced psychological safety, workforce exclusion, burnout, underutilisation of expertise and poorer health outcomes.

The framework further proposes that nurses occupy a unique structural position within healthcare systems because of their sustained engagement with patients, workforce systems and organisational processes. This positioning creates both visibility of inequities and professional responsibility for structural intervention.

Importantly, the framework argues that traditional models of nursing advocacy focused primarily on individual patient protection are insufficient to address inequities embedded within institutional systems. Instead, structural advocacy is required, incorporating governance accountability, workforce reform, structural competency education, policy engagement, equity‐integrated clinical governance and anti‐racist organisational leadership.

Existing structural competency, health equity, anti‐racism and culturally safe care frameworks have made important contributions by increasing awareness of structural inequities, promoting culturally responsive practice and strengthening organisational reflection on equity (Braveman et al. 2022; Metzl and Hansen 2014; Moorley et al. 2020). The NSERF complements and extends these approaches by moving beyond conceptual recognition of structural inequities to provide a nurse‐led implementation framework for governance, workforce, education and policy reform. Rather than focusing primarily on recognising structural inequities, the framework provides an implementation‐oriented roadmap that integrates governance accountability, workforce reform, equity‐integrated clinical governance, policy engagement, organisational leadership and measurable accountability mechanisms into a coordinated model for sustained structural change. Ultimately, the NSERF provides a practical, nurse‐led framework for translating structural equity principles into measurable organisational and system‐level reform, with the potential to improve patient safety, workforce sustainability, healthcare equity, organisational trust and culturally safe healthcare systems.

## 3 Structural Racism Across Healthcare Systems

Structural racism manifests across interconnected levels of healthcare systems, including policy, organisational governance, workforce integration, clinical practice, professional advancement and organisational culture. These domains are mutually reinforcing. Inequitable policies shape organisational priorities; organisational cultures influence professional norms; professional norms shape clinical decision‐making; and workforce inequities influence who holds authority, whose expertise is recognised and whose concerns are acted upon. (see Table S1 for a summary of these system‐level mechanisms).

Within healthcare systems, structural racism contributes to disparities in access to care, delayed escalation of treatment, inequitable referral pathways, disparities in pain management and reduced quality of care for racialised populations (Hamed et al. 2022). Structural inequities also shape workforce systems through inequitable credential recognition, discriminatory recruitment practices, underrepresentation within leadership structures, and exclusionary organisational cultures affecting internationally qualified and racialised healthcare professionals (Kurup et al. 2023; Kurup et al. 2024b). For example, inequitable staffing algorithms, discriminatory triage protocols and inconsistent access to interpreter services illustrate how ostensibly neutral organisational policies can systematically disadvantage racialised patients and staff (Braveman et al. 2022).

These inequities are frequently reproduced through organisational processes that appear institutionally neutral while continuing to generate patterned disadvantage. Consequently, structural racism should be understood not as isolated interpersonal incidents, but as a systemic governance and accountability challenge embedded within healthcare structures.

### 3.1 Patient‐Level Consequences

At the patient level, structural racism contributes to unequal access to healthcare services, differential treatment decisions and disparities in health outcomes. Hall et al. (2015) identified widespread implicit racial and ethnic bias among healthcare professionals and demonstrated associations between bias, communication quality, treatment recommendations, adherence and patient outcomes. (see Table S2 for a summary of patient‐, workforce‐ and system‐level consequences).

Evidence further demonstrates that racialised populations frequently experience dismissal of symptoms, barriers to access, delayed intervention and reduced quality of care (Hamed et al. 2022). These inequities become particularly visible during crises and emergencies. Rossen (2021) highlighted how COVID‐19 disparities reflected structural conditions affecting exposure, healthcare access and system responsiveness rather than individual behaviours alone.

If structural racism produces predictable and preventable harms, equity must be managed with the same seriousness afforded to medication safety, infection prevention and clinical governance. Healthcare organisations should therefore routinely monitor race‐stratified indicators relating to access, referral pathways, escalation patterns, discharge outcomes and patient‐reported experience measures to identify and address inequities in real time.

### 3.2 Workforce‐Level Consequences

Structural racism also shapes healthcare workforce experiences through inequitable recruitment, credentialing, role allocation, professional development, disciplinary practices and promotion pathways. Evidence demonstrates recurrent patterns of deskilling and underutilisation of expertise among internationally qualified and racialised nurses, alongside discriminatory assumptions regarding competence and limited access to professional progression opportunities (Kurup et al. 2024a).

Evidence indicates that organisational policies, credential recognition processes and workforce structures may limit the effective utilisation of internationally educated nurses' skills and expertise following migration. Divergent understandings of specialist nursing roles, exclusionary workplace cultures and organisational hierarchies have been associated with inequitable recruitment practices, restricted career progression, role mismatch and under‐recognition of professional expertise among migrant and racialised nurses (Kurup et al. 2026a, 2026b).

Workplace racism additionally affects retention, wellbeing and workforce sustainability. Mostafa et al. (2025) identified recurring experiences of exclusionary workplace cultures, insufficient organisational support, emotional exhaustion and psychological harm among racialised healthcare professionals. These workforce inequities are not peripheral organisational concerns; they directly influence team functioning, workforce stability, patient safety and healthcare quality. Collectively, these findings indicate that structural racism erodes nurses' resilience and professional wellbeing, with direct consequences for retention and long‐term workforce sustainability across healthcare organisations.

### 3.3 Organisational Governance and Accountability

Structural racism is sustained through organisational cultures and governance systems that privilege dominant norms, reinforce hierarchical power structures and inadequately address inequity within leadership and decision‐making structures (Moorley et al. 2020). Equity initiatives are frequently treated as supplementary or symbolic rather than embedded within core governance systems, leaving reform efforts vulnerable to fragmentation and performative implementation.

This paper adopts a clear position that equity must be integrated within healthcare governance. Healthcare organisations should incorporate equity as a standing component of clinical governance and quality systems, require equity impact assessments for service redesign and ensure nursing leadership, including leadership from racialised professionals, holds formal decision‐making authority.

Equity‐integrated clinical governance should therefore become a core organisational expectation rather than an optional diversity initiative. This may include routine race‐stratified monitoring of patient safety indicators, escalation patterns, referral rates, waiting times, discharge outcomes, workforce progression and patient‐reported experience measures. Organisations should additionally implement equity dashboards, anti‐racism accountability structures and executive‐level oversight of workforce and patient equity indicators.

### 3.4 Ethical and Professional Foundations for Nurse‐Led Advocacy

Addressing structural racism aligns directly with nursing's ethical obligations, including justice, beneficence and respect for dignity (International Council of Nurses 2023). Justice requires active engagement with systemic inequities that produce unequal outcomes, while beneficence requires preventing foreseeable harms arising from institutional barriers and exclusionary systems (see Table S3 for a summary of the ethical mandate, practice insight and workforce presence underpinning this interface).

Contemporary nursing identity increasingly extends beyond bedside care towards leadership, governance and system transformation. Neutrality in the face of patterned inequity is incompatible with ethical nursing practice. Advocacy must therefore be understood not solely as individual patient protection, but as a structural and professional responsibility extending into organisational reform, workforce policy and healthcare governance (Pitcher et al. 2025).

### 3.5 From Individual Advocacy to Structural Advocacy

Traditional nursing advocacy has historically focused on protecting individual patients, promoting safety and facilitating access to care. Although these functions remain essential, they are insufficient to address inequities embedded within institutional systems and policy environments. (see Table S4 for a comparison of individual and structural advocacy across key domains).

Structural advocacy involves deliberate engagement with the systemic conditions shaping healthcare access, workforce experiences, professional advancement and organisational accountability. This includes participation in governance structures, workforce policy reform, education design, service planning and broader health policy engagement.

Structural advocacy additionally requires explicit attention to workforce equity, including equitable credential recognition, inclusive progression pathways and organisational accountability for workplace racism affecting internationally qualified and racialised nurses (Mostafa et al. 2025).

### 3.6 Strategic Directions for Nurse‐Led Structural Reform

Advancing equity‐driven reform requires a coordinated nursing agenda spanning governance, workforce policy, education, organisational culture and public policy advocacy. Nursing representation within leadership and decision‐making structures must be strengthened because structural racism is reproduced through institutional policy, resource allocation and professional hierarchies. Without meaningful nursing authority within governance systems, equity initiatives remain vulnerable to fragmentation and symbolic compliance rather than measurable reform. (see Table S5 for a summary of nurse‐led reform pathways across governance, education, workforce policy, advocacy and organisational culture).

Nursing education should additionally embed structural competency, equipping nurses to identify and respond to structural racism, institutional inequities, cultural unsafety and organisational determinants of inequity rather than limiting equity education to interpersonal cultural awareness alone (Braveman et al. 2022; Moorley et al. 2020). This shift is essential for preparing nurses to engage with the systemic and organisational conditions that shape both patient outcomes and workforce experiences.

Workforce reform must include equitable credential recognition, transparent progression pathways, anti‐racism workplace protections and meaningful integration of internationally qualified nurses. Emerging evidence demonstrates that workforce inequities are frequently sustained through institutional credentialing systems, inconsistent specialty skill recognition and exclusionary organisational cultures, reinforcing the need for governance‐level workforce reform and accountability mechanisms (Kurup et al. 2025). Inequitable workforce systems diminish psychological safety, constrain expertise utilisation and undermine organisational credibility regarding equity commitments.

Importantly, examples of positive structural reform are increasingly emerging internationally. These include culturally safe healthcare frameworks, Indigenous health governance models, anti‐racism workforce initiatives, inclusive leadership programmes, equity‐focused quality improvement systems and structured transition programmes supporting internationally qualified nurses. Some healthcare organisations have additionally implemented race‐stratified outcome monitoring, equity dashboards and organisational accountability structures aimed at reducing inequities in both workforce experiences and patient care. Concrete mechanisms for embedding this agenda include establishing equity‐integrated clinical governance committees with authority to mandate equity impact assessments for service redesign; implementing workforce equity dashboards that track recruitment, promotion and attrition by racial background and migration pathway; embedding leadership accountability through equity‐linked performance indicators for executive and nursing leadership; conducting culturally safe quality improvement audits of clinical pathways and escalation processes; and engaging in policy advocacy through professional nursing bodies to embed anti‐racism standards within accreditation and regulatory frameworks. Structured mentorship and sponsorship programmes for internationally qualified and racialised nurses further support equitable career progression and workforce retention.

Lasting transformation ultimately requires organisational cultures that explicitly prioritise anti‐racism accountability, equity‐integrated governance, inclusive professional norms and sustained leadership engagement consistent with nursing's ethical obligations to justice and equity (Braveman et al. 2022; Moorley et al. 2020).

## 4 Future Research

Although the NSERF provides a conceptual roadmap for addressing structural racism through nurse‐led governance, workforce, education and policy reform, its effectiveness now requires empirical evaluation. Future research should validate the framework across diverse healthcare settings and examine its impact on patient outcomes, nurses' wellbeing, psychological safety, workforce retention, organisational culture and governance accountability. Implementation science approaches, longitudinal studies and the development of validated structural equity indicators are needed to determine how the framework can be effectively operationalised, monitored and adapted across different national and international healthcare systems.

## 5 Conclusion

Structural racism remains a persistent and preventable determinant of healthcare inequity that compromises patient safety, workforce sustainability, organisational trust and health outcomes globally. Addressing structural racism is not peripheral to nursing practice but central to nursing's ethical, professional and leadership responsibilities. Left unaddressed, structural racism also continues to erode nurses' wellbeing, resilience and retention, threatening the long‐term sustainability of the nursing workforce upon which safe, equitable care depends.

The NSERF advances a structural advocacy approach positioning nurses as critical agents of governance accountability, workforce reform and equity‐driven healthcare transformation. Moving beyond interpersonal advocacy towards sustained structural reform is essential for developing healthcare systems that are equitable, culturally safe, accountable and sustainable for both patients and healthcare professionals. In doing so, this paper extends beyond existing descriptive and conceptual discussions of structural racism by offering a practical, integrated framework that nurse leaders, educators, researchers and policymakers can apply to drive measurable structural reform.

## Author Contributions

**Deepa Rijal:** conceptualization, investigation, methodology, validation, formal analysis, data curation. **Chanchal Kurup:** conceptualization, methodology, investigation, validation, visualization, formal analysis, data curation, supervision, project administration.

## Funding

The authors have nothing to report.

## Ethics Statement

The authors have nothing to report.

## Consent

The authors have nothing to report.

## Conflicts of Interest

The authors declare no conflicts of interest.

## Supporting information


**Table S1:** System‐level mechanisms of structural racism in healthcare systems.
**Table S2:** Observable consequences of structural racism in healthcare systems.
**Table S3:** Professional nursing interface with structural racism.
**Table S4:** Advocacy continuum—from individual to structural advocacy.
**Table S5:** Nurse‐led structural reform pathways.

## Data Availability

The datasets used and/or analysed during the current study are available from the corresponding author on reasonable request.

## References

[nop270754-bib-0001] Braveman, P. A. , E. Arkin , D. Proctor , T. Kauh , and N. Holm . 2022. “Systemic and Structural Racism: Definitions, Examples, Health Damages, and Approaches to Dismantling.” Health Affairs 41, no. 2: 171–178. 10.1377/hlthaff.2021.01394. 35130057

[nop270754-bib-0002] Hall, W. J. , M. V. Chapman , K. M. Lee , et al. 2015. “Implicit Racial/Ethnic Bias Among Health Care Professionals and Its Influence on Health Care Outcomes: A Systematic Review.” American Journal of Public Health 105, no. 12: e60–e76. 10.2105/ajph.2015.302903. PMC4638275 26469668

[nop270754-bib-0003] Hamed, S. , H. Bradby , B. M. Ahlberg , and S. Thapar‐Björkert . 2022. “Racism in Healthcare: A Scoping Review.” BMC Public Health 22, no. 1: 988. 10.1186/s12889-022-13122-y. 35578322 PMC9112453

[nop270754-bib-0004] International Council of Nurses . 2023. “The Global Nursing Workforce and the Future of Healthcare.” www.icn.ch/sites/default/files/2023‐07/ICN_Recover‐to‐Rebuild_report_EN.pdf.

[nop270754-bib-0005] Kisa, A. , and S. Kisa . 2025. “Structural Racism as a Fundamental Cause of Health Inequities: A Scoping Review.” International Journal for Equity in Health 24, no. 1: 257. 10.1186/s12939-025-02644-7. 41063084 PMC12506018

[nop270754-bib-0006] Kurup, C. , V. Betihavas , A. Burston , and E. Jacob . 2023. “Strategies Employed by Developed Countries to Facilitate the Transition of Internationally Qualified Nurses Specialty Skills Into Clinical Practice: An Integrative Review.” Nursing Open 10, no. 12: 7528–7543. 10.1002/nop2.2023. 37794722 PMC10643820

[nop270754-bib-0007] Kurup, C. , A. S. Burston , V. Betihavas , and E. R. Jacob . 2024a. “Internationally Qualified Nurses' Perspectives on Transitioning Specialty Skills Within Australia: A Content Analysis.” Nursing Open 11, no. 9: 1–17. 10.1002/nop2.70032. PMC11386253 39252497

[nop270754-bib-0008] Kurup, C. , A. S. Burston , V. Betihavas , and E. R. Jacob . 2024b. “The Perspectives of Internationally Qualified Nurses Regarding Their Specialty Skill Transition to Australia: A Cross‐Sectional Survey.” Journal of Advanced Nursing 80, no. 5: 1868–1881. 10.1111/jan.15952. 37975414

[nop270754-bib-0009] Kurup, C. , A. S. Burston , V. Betihavas , and E. R. Jacob . 2025. “Exploring the Utilisation of Internationally Qualified Nurses' Specialty Skills: Analysis of Recruiting Managers' View Points.” Nursing Open 12, no. 3: e70184. 10.1002/nop2.70184. 40102055 PMC11919484

[nop270754-bib-0010] Kurup, C. , A. S. Burston , V. Betihavas , and E. R. Jacob . 2026a. “Equity in Action: Disrupting Systemic Barriers to Specialty Skill Utilisation Among Internationally Qualified Nurses in Australia.” Nursing Open 13, no. 4: e70541. 10.1002/nop2.70541. 41964306 PMC13069174

[nop270754-bib-0011] Kurup, C. , A. S. Burston , V. Betihavas , and E. R. Jacob . 2026b. “How Divergent Views of Specialist Nurse Status Affect Iqns' Skill Utilisation in Australia.” Journal of Nursing Management 2026, no. 1: 5922358. 10.1155/jonm/5922358. 41647686 PMC12868908

[nop270754-bib-0012] Metzl, J. M. , and H. Hansen . 2014. “Structural Competency: Theorizing a New Medical Engagement With Stigma and Inequality.” Social Science & Medicine 103: 126–133. 10.1016/j.socscimed.2013.06.032. 24507917 PMC4269606

[nop270754-bib-0013] Moorley, C. , P. Darbyshire , L. Serrant , J. Mohamed , P. Ali , and R. De Souza . 2020. “Dismantling Structural Racism: Nursing Must Not Be Caught on the Wrong Side of History.” Journal of Advanced Nursing 76, no. 10: 2450–2453. 10.1111/jan.14469. 32692444

[nop270754-bib-0014] Mostafa, I. , J. Onwumere , and L. Wood . 2025. “A Systematic Review and Thematic Synthesis of Healthcare Professionals' Experiences of Racism in the Workplace and the Support They Receive.” Journal of Workplace Behavioral Health: 1–29. 10.1080/15555240.2025.2519298.

[nop270754-bib-0015] Pitcher, C. , A. Slemon , and M. Danda . 2025. “Reconceptualizing Advocacy: A Novel Model for Nursing Theory and Practice.” Nursing Inquiry 32, no. 3: e70036. 10.1111/nin.70036. 40618399 PMC12229263

[nop270754-bib-0016] Rossen, L. M. 2021. “Disparities in Excess Mortality Associated With COVID‐19—United States, 2020.” Morbidity and Mortality Weekly Report 70, no. 33: 1114–1119. 10.15585/mmwr.mm7033a2. 34411075 PMC8375709

[nop270754-bib-0017] Williams, D. R. , J. A. Lawrence , and B. A. Davis . 2019. “Racism and Health: Evidence and Needed Research.” Annual Rreview of Public Health 40, no. 1: 105–125. 10.1146/annurev-publhealth-040218-043750. PMC6532402 30601726

