# Supplementary material for: Structural Racism in Healthcare: Nurses' Ethical and Professional Responsibility to Lead Structural Reform
Source: Nurs Open. 2026 Aug 26;13(9):e70754. doi: 10.1002/nop2.70754 (PMC13519522; doi:10.1002/nop2.70754)
Supplement: Supplementary file 1 [file NOP2-13-e70754-s001.docx]

# **Supplementary Material**

Supplementary Tables 1–5 present the underlying constructs of the Nurse-Led Structural Equity Reform Framework (NSERF) shown in Figure 1, detailing the system-level mechanisms, observable consequences, professional nursing interface, advocacy continuum, and nurse-led reform pathways that comprise the framework.

***Supplementary Table 1: System-Level Mechanisms of Structural Racism in Healthcare Systems***

| **Policy structures** | **Organisational governance** | **Workforce integration** | **Professional hierarchies** | **Institutional culture** |
| --- | --- | --- | --- | --- |
| Credential recognition pathways | Leadership representation | Recruitment practices | Scope of practice allocation | Normative decision-making |
| Service configuration | Equity governance mechanisms | Role allocation | Career progression pathways | Inclusion/exclusion norms |
| Funding priorities | Accountability systems | Professional development access | Knowledge legitimacy | Informal power structures |

***Supplementary Table 2: Observable Consequences of Structural Racism in Healthcare Systems***

| **Patient-level outcomes** | **Workforce-level outcomes** | **System-level outcomes** |
| --- | --- | --- |
| Unequal access to care | Deskilling of internationally qualified nurses (IQNs) | Reduced service capability |
| Differential treatment | Underutilisation of expertise | Workforce inefficiency |
| Delayed diagnosis | Psychological insecurity | Increased attrition |
| Poorer outcomes | Restricted progression | Inequitable service delivery |

***Supplementary Table 3: Professional Nursing Interface with Structural Racism***

| **Ethical mandate** | **Practice insight** | **Workforce presence** |
| --- | --- | --- |
| Justice | Proximity to care systems | Largest global workforce |
| Advocacy | Identification of inequities | Cross-system engagement |
| Beneficence | Implementation insight | Policy engagement potential |

***Supplementary Table 4: Advocacy Continuum — From Individual to Structural Advocacy***

| **Individual advocacy** | **Structural advocacy** |
| --- | --- |
| Patient rights | Workforce policy reform |
| Access to services | Credential recognition reform |
| Clinical safety | Governance redesign |
| Care coordination | Resource allocation equity |
| Immediate care needs | Institutional accountability |

***Supplementary Table 5: Nurse-Led Structural Reform Pathways***

| **Governance** | **Education** | **Workforce policy** | **Advocacy** | **Organisational culture** |
| --- | --- | --- | --- | --- |
| Leadership representation | Structural competency | Fair credential recognition | Policy engagement | Anti-racism accountability |
| Equity in committees | Equity-integrated curricula | Transparent progression | Service redesign | Inclusive norms |
| Decision-making authority | Critical policy literacy | Skill utilisation pathways | Legislative influence | Cultural safety |
